# Molecular Iodine—An Expedient Reagent for Oxidative Aromatization Reactions of α,β-Unsaturated Cyclic Compounds

**DOI:** 10.3390/molecules14125308

**Published:** 2009-12-16

**Authors:** Malose Jack Mphahlele

**Affiliations:** Department of Chemistry, College of Science, Engineering and Technology, University of South Africa, P.O. Box 392, Pretoria 0003, South Africa; E-Mail: mphahmj@unisa.ac.za; Tel. +27-12-429-8805; Fax: +27-12-429-8549

**Keywords:** iodine, oxidative aromatization, α,β-unsaturated cyclic compounds

## Abstract

Prompted by the scant attention paid by published literature reviews to the applications of molecular iodine in oxidative aromatization reactions, we decided to review methods developed to-date involving iodine as an oxidant to promote aromatization of α,β-unsaturated cyclic compounds.

## 1. Introduction

Aromatization of substituted cyclohexenones to the corresponding phenol or phenyl ether derivatives has attracted a great deal of attention for a long time. Catalytic dehydrogenation of substituted 2-cyclohexen-1-one derivatives using 5% palladium-carbon in high boiling hydrocarbon solvents, for example, previously afforded the corresponding phenolic systems in high yields [1]. Under similar reaction conditions, substituted 2-cyclohexenone derivatives were found to undergo disproportionation leading to reduced yields of the target phenolic or phenyl ether derivatives [2]. In another development, copper(II) bromide/lithium bromide mixture in boiling acetonitrile was applied to 2-cyclohexen-1-ones and their fused derivatives to afford 75–85% of the corresponding phenolic compounds [3]. This reaction, which is believed to proceed by halogenation of the homoannular enol form of the conjugated carbonyl group, was found to occur with conservation of ring junction stereochemistry and without halogenation α to the nonconjugated carbonyl group. Dehydrogenation of 4-oxo-4,5,6,7-tetrahydrobenzofuran-2-carboxylic acid and its methyl ester derivative with copper(II) bromide (CuBr_2_) in refluxing methanol also afforded the corresponding 4-hydroxy-2,3-dihydrobenzofuran-2-carboxylic acid derivatives [4]. Despite the observed trans-esterification or esterification, the application of CuBr_2_ was found to be more effective than the use of sulfur at 250 ºC or 2,3-dichloro-5,6-dicyano-1,4-benzoquinone (DDQ) in benzene under reflux [4]. Homogenous transition metal complex such as rhodium trichloride trihydrate (RhCl_3_·3H_2_O) was also found to induce oxidative aromatization and remote double bond migration of alkenylcyclohexenones and unsaturated imines to afford substituted phenols and aniline derivatives [5]. Treatment of ω-alkenyl substituted cyclohexenone-1,3-diones with RhCl_3_·3H_2_O in methanol or ethanol afforded substituted resorcinols [6]. Under similar reaction conditions, related enol ethers afforded dienones. Vanadium-catalyzed oxidative aromatization of α,β-unsaturated cyclohexenones using VO(OR)Cl_2_ in refluxing alcohols also afforded aryl ether derivatives [7]. An efficient catalytic oxidative aromatization of 2-cyclohexenones involving a combination of a commercially available ligand-free vanadium catalyst (VOSO_4_), a bromide source (Bu_4_NBr or HBr), and an acid (trifluoroacetic acid) under atmospheric oxygen or air recently afforded the corresponding phenol derivatives [8] Although heterogenous or homogenous metal–catalyzed aromatization of substituted cyclohexenones to the corresponding phenols or phenol ethers is a well established procedure, it involves severe reaction conditions accompanied by prolonged reaction times. 

The oxidative potential of iodine has been exploited over the years in the synthesis of novel aromatic and heteroaromatic compounds that may possess some biological activity or serve as building blocks for the synthesis of derivatives with potential biological applications. The naturally occurring olivetol (**1a)** and the antifungal antibiotic DB2073 (**1b**), for example, were synthesized before from substituted resorcinols, prepared in turn from the corresponding 1,3-cyclohexanediones using iodine in refluxing methanol [9]. 4-Methoxy-2-phenylquinoline (**2a**) and its 4-methoxy-2-(3,4-methylenedioxyphenyl)quinoline analogue **2b**, which are plant-based [10] are readily accessible in the laboratory *via* iodine-mediated oxidative aromatization of the corresponding 2-aryl-1,2,3,4-tetrahydroquinolin-4-ones [11]. These alkoxyquinoline derivatives, which are reported to exhibit inhibitory activity against *Mycobacterium tuberculosis* H_37_Rv [12] can also be accessible from 2-aryl-1,2,3,4-tetrahydroquinolin-4-ones using thallium(III) nitrate [13] or [hydroxyl(tosyloxy)iodo]benzene [14] in trimethyl orthoformate in the presence of catalytic amount of perchloric acid or using FeCl_3_.6H_2_O in methanol [15]. The use of potentially explosive perchloric acid and/ or environmentally unfriendly metallic reagents that are not easy to obtain represent some of the drawbacks of these methods. 

**Figure 1:**
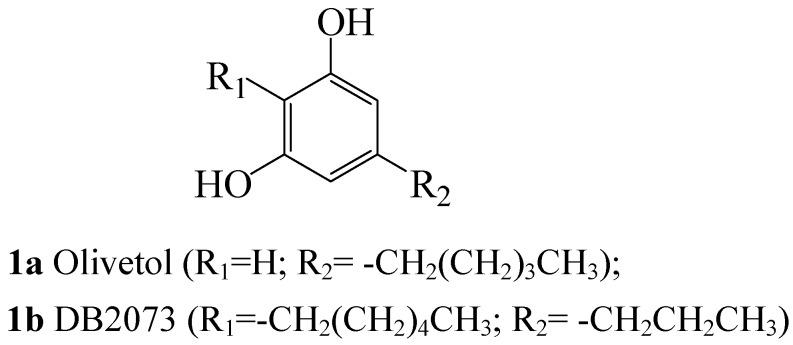
Structures of olivetol **1a** and DB2073 **1b**.

**Figure 2:**
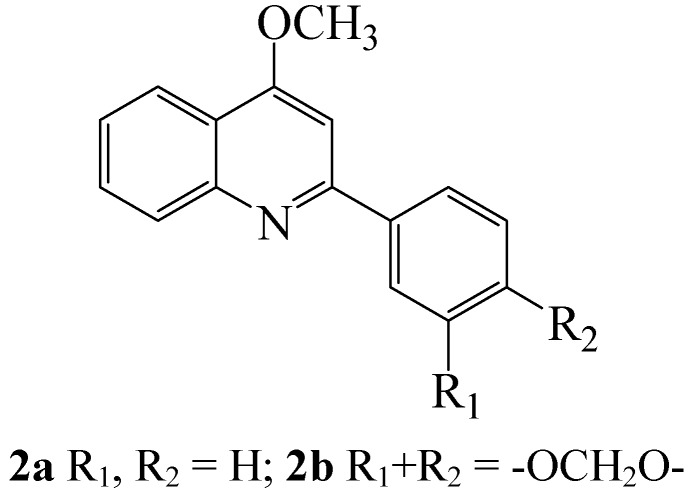
Structures of naturally occurring 2-aryl-4-methoxyquinolines.

In recent years, molecular iodine has received considerable attention as an inexpensive, non-toxic, readily available oxidant to promote aromatization of cyclohexenone derivatives and their heterocyclic analogues. Despite the growing applications of iodine as an expedient oxidizing agent to promote oxidative aromatization of α,β-unsaturated cyclic compounds such examples feature less or not at all in literature reviews on the applications of iodine in various chemical transformation as a Lewis acid catalyst, electrophile or oxidant [16,17,18,19]. We address this need in the present review by focusing primarily on the application of molecular iodine as an oxidizing agent to effect aromatization of α,β-unsaturated cyclic compounds and their heterocyclic analogues.

## 2. Iodine as an Oxidant

### 2.1. Iodine-alcohol–mediated aromatization of cyclohexenone derivatives

**Scheme 1:**
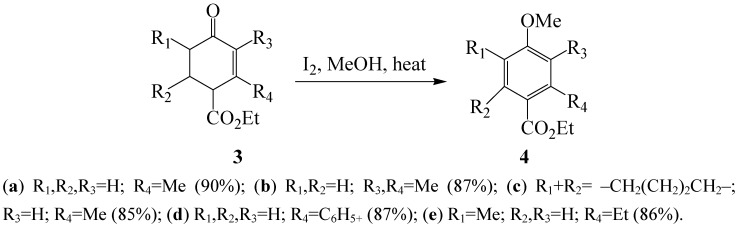
Iodine-promoted oxidative aromatization of Hagemann’s esters.

The use of molecular iodine as an oxidant to promote aromatization of cyclohexenone derivatives was first reported in 1980 by Tamura and Yoshimoto [20]. These authors subjected series of cyclohexenones to iodine in refluxing methanol to afford variously substituted anisole derivatives. Their methodology was later applied by Kotnis on Hagemann’s esters **3** to afford substituted *p*-methoxybenzoates **4**, which are building blocks for several marine natural products (Scheme 1) [21].

**Scheme 2:**
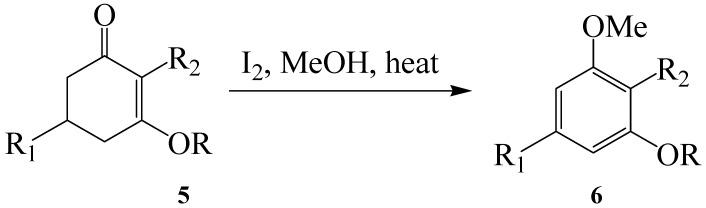
Iodine-promoted oxidative aromatization of cyclohexane-1,3-diones to anisole derivatives.

In another development involving the use of iodine-methanol reaction mixture, Kotnis transformed a series of cyclohexane-1,3-dione derivatives **5** (for **5e**; R=Ac) to substituted resorcinols **6** (Scheme 2) [9]. The only mechanistic suggestion was that 1,4-addition–elimination of methanol to the enol form of the cyclohexadione system takes place as a first step of the reaction. The author also used some of the prepared resorcinols as precursors for the synthesis of olivetol **2a** and the antifungal antibiotic DB2073 **2b** [9].

**Scheme 3:**
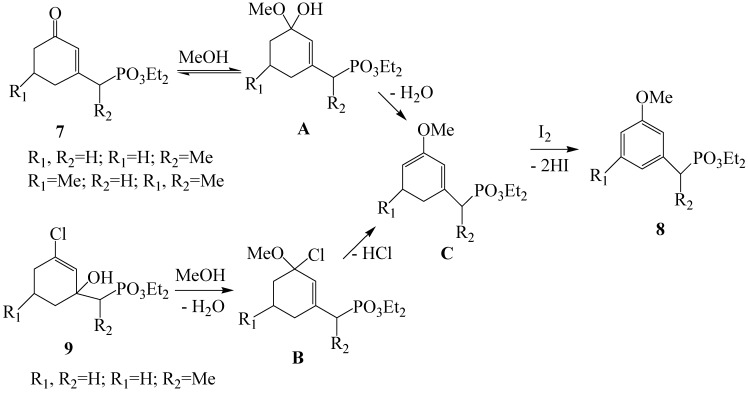
Iodine-promoted oxidative aromatization of 3-phosphonomethylcyclohexenones.

**Scheme 4:**
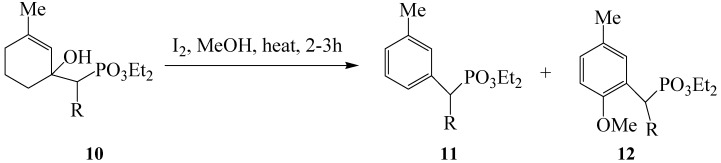
Iodine-mediated aromatization of diethyl 1-(1-hydroxy-3-methylcyclohex-2-enyl)alkylphosphonates.

**Scheme 5:**
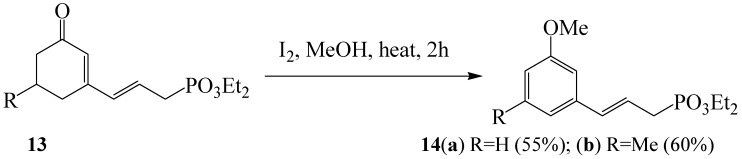
Iodine-mediated aromatization of ketophosphonates **15**.

Similar reaction conditions to those previously employed by Kotnis were later applied to 3-(phosphonoalkyl)cyclohexenones **7** to afford a series of novel anisole derivatives **8** substituted at the 3-position with alkylphosphonate group (Scheme 3) [22]. The observed results were interpreted as a consequence of an initial 1,2- (independent of 1,4-) addition of methanol, followed by dehydration and iodine-promoted oxidative aromatization. The same products were also obtained from the corresponding 3-chlorocyclohexenols **9**, presumably *via* the acid catalyzed S_N_2’ displacement of the allylic alcohol by methanol. Elimination of HCl would lead to the same cyclohexadiene derivative as obtained from the cylohexenone derivative and is followed by the iodine–promoted aromatization. Under similar reaction conditions applied to **7**, diethyl 1-(1-hydroxy-3-methylcyclohex-2-enyl)ethylphosphonates **10** afforded the expected 3-methylbenzylphosphonates **11,** as well as their 6-methoxy derivatives **12** *albeit* in low yields (Scheme 4) [22]. The reaction was also found to work well with cyclohexenone derivatives **13** bearing allylphosphonate moiety at the 3-position to afford novel 3-substituted anisole derivatives **14** in moderate yields (Scheme 5) [23].

**Scheme 6:**
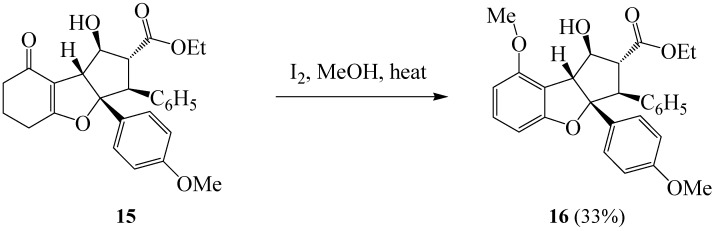
Iodine-methanol–mediated aromatization of dihydrofuran derivative **11**.

**Scheme 7:**
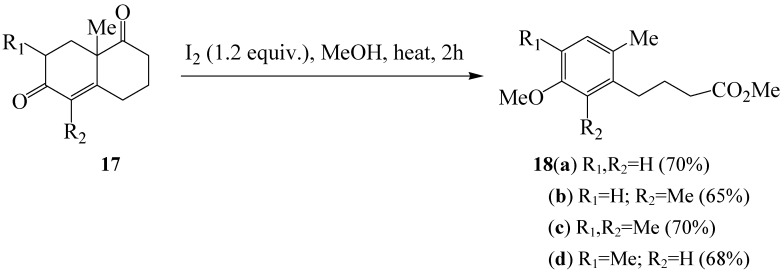
Iodine-methanol–mediated aromatization of cyclic diones.

Dihydrofuran **15** was previously subjected to iodine in refluxing methanol to afford the aromatized derivative **16**, which is an analogue of the naturally occurring rocaglamide (Scheme 6) [24]. A one-pot iodine-methanol–mediated aromatization of cyclic diones **17** and subsequent fragmentation to anisole derivatives **18** has also been reported before (Scheme 7) [25].

**Scheme 8:**
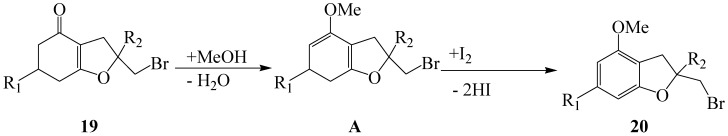
Iodine-mediated aromatization of 2-halogenomethyl-3,5,6,7-tetrahydrobenzofuran-4-ones.

**Scheme 9:**
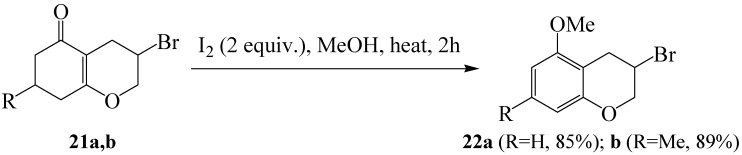
Iodine-mediated aromatization of 3-bromo-2,3,4,6,7,8-hexahydro-1-benzopyran-5-ones.

The oxidative properties of iodine were recently exploited to effect aromatization of 2-bromomethyl-3,5,6,7-tetrahydrobenzofurans **19** to afford the corresponding 2-bromomethyl-4-methoxy-2,3-dihydrobenzofurans **20** (Scheme 8) [26]. Under similar reaction conditions, the analogous 2-bromo-2,3,4,6,7,8-hexahydro-1-benzopyran-5-ones **21** (R=H, Me) afforded the corresponding 3-bromo-5-methoxy-3,4-dihydrobenzopyran derivatives **22a** (R=H) and **22b** (R=Me) in 85% and 89% yields, respectively (Scheme 9). Moreover, the combined electrophilic and oxidative properties of iodine were also exploited to effect direct one-pot iodocyclization of 2-allylcyclohexane-1,3-diones and subsequent *in situ* oxidative aromatization of the resulting 2-iodomethyl-2,4,5,6-tetrahydrobenzofuran-4-ones to afford 2-iodomethyl-4-methoxy-2,3-dihydrobenzofurans [26].

**Scheme 10:**
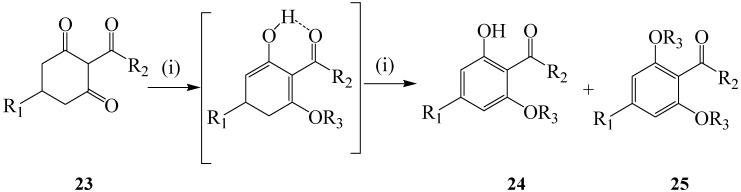
Iodine-promoted oxidative aromatization of 2-acyl- and 2-propionylcyclohexane-1,3-diones.

Iodine in refluxing alcohols was also employed before by Kim and co-workers on 2-acyl- and 2-propionylcyclohexane-1,3-diones **23** (Scheme 10) [27]. The 3-alkoxy-1-hydroxyacetophenone derivatives **24a-d** were formed exclusively from the acetyl- and propionyl substituted starting materials **23a** and **23d**. The absence of dimethoxy derivatives in the case of acetyl- and propionyl substituted starting materials **23a** and **23d** was attributed to strong intramolecular hydrogen bonding that would prevent conversion of the hydroxyl group into methoxy group. Mixtures of mono- **24** and dimethoxy derivatives **25** were isolated when the benzoyl or carbomethoxycyclohexane-1,3-diones **23e,f**, **h** were used as substrates. In the latter instance, the authors attributed the formation of both monomethoxy and dimethoxy derivatives to be a consequence of the weakly hydrogen bonding 2-benzoyl and 2-carbomethoxy groups. However, these authors could not account for the formation of both monomethoxy and dimethoxy derivatives when the 5-methyl substituted 2-acetylcyclohexane-1,3-dione **23g** was used as a substrate.

**Scheme 11:**
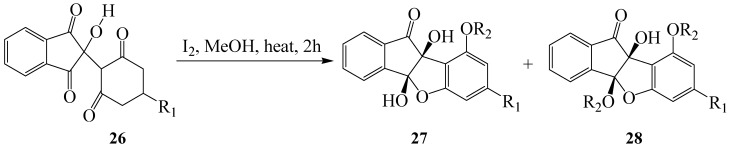
Iodine-methanol oxidative aromatization of benzo[*b*]indeno[2.1-*d*]furanone derivatives.

Treatment of benzo[*b*]indeno[2.1-*d*]furanone derivatives **26** with iodine (2 equiv.) in refluxing methanol afforded anisole derivatives **27** (minor) and **28** (major), respectively (Scheme 11) [28]. The formation of methyl derivatives **28** was rationalized as a consequence of initial acid-catalyzed dehydration of **27** to form a cyclic oxonium intermediate, which then undergoes addition of methanol [25]. The proposed mechanism was proven in a follow up study involving selective methylation of systems **27** to **28** using iodine-methanol mixture and the methylated derivatives were found to be formed selectively under prolonged heating conditions (13–36 h) [29].

**Scheme 12:**
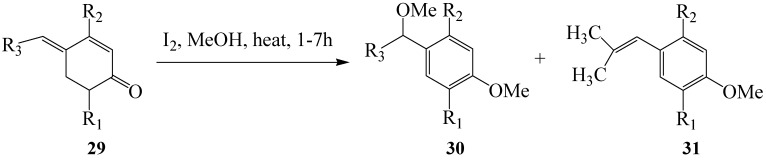
Iodine-methanol promoted oxidative aromatization of 4-alkylidene-2-cyclohexen-1-ones.

Kim and coworkers also employed iodine in refluxing methanol to effect oxidative aromatization of 4-alkylidene-2-cyclohexen-1-ones **29** to afford the corresponding anisole derivatives (Scheme 12) [30]. The mechanism of this reaction which was also confirmed using iodine (1.1 equiv.) on **29b** in deuterated methanol to afford deuterated analogue of **30b** (OCD_3_ in place of OMe) in 32% yield is believed to involve initial conjugate addition of methanol to the exo-methylene moiety followed by attack of the carbonyl carbon by methanol to generate a hemiketal derivative. Dehydration of the latter then occurs followed by iodine-promoted oxidative aromatization to yield the anisole derivatives. The fully conjugated systems **31f** and **31g** formed as minor products from substrates **29f** and **29g** are presumably the consequence of slow expulsion of methanol from the dimethoxy products. Kim’s group also subjected 2-methylene-2-cyclohexenones to iodine in alcohol (methanol or ethanol) to afford series of novel anisole derivatives [31].

**Scheme 13:**
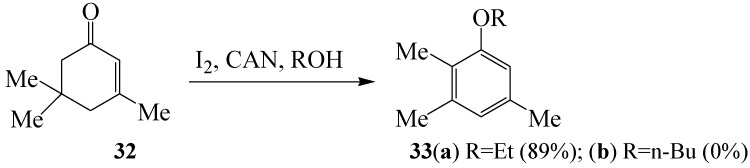
Iodine-CAN promoted oxidative aromatization of trimethyl-2-cyclohexen-1-ones.

2-Cyclohexen-1-one and its 3-methyl derivative with iodine-cerium(IV) ammonium nitrate mixture in alcohol (methanol, ethanol, 1-propanol, 2-propanol) previously afforded the corresponding alkyl phenyl ethers in moderate to high yields [32]. Cerium(IV) ion is believed to coordinate with the carbonyl oxygen to facilitate attack by alcohol leading to enolization, which in turn facilitates iodine-mediated oxidative aromatization to afford alkyl phenylether derivatives. 3,5,5-Trimethyl-2-cyclohexen-1-one **32** was found to undergo methyl shift upon treatment with iodine-CAN in ethanol or n-butanol to afford the corresponding 3,4,5-trimethyl substituted phenylether derivatives **33a** and **b**, in 89% and 90% yields, respectively (Scheme 13) [32].

Iodine represents a relatively less expensive reagent for oxidative aromatization of cyclohexenone moiety than metal–catalyzed aromatization of substituted cyclohexenones to the corresponding phenol ethers or phenols [1,2,3,4,5,6,7,8]. It is also superior to the use of 2,3-dichloro-5,6-dicyano-1,4-benzoquinone (DDQ) in dioxane, which was previously employed to dehydrogenate 5-acetyl-4-oxo-4,5,6,7-tetrahydrobenzofuran and methyl-4-oxo-4,5,6,7-tetrahydrobenzofuran-5-carboxylate [33].

### 2.2. Iodine-methanol–mediated aromatization of 4-quinolone derivatives

**Scheme 14:**
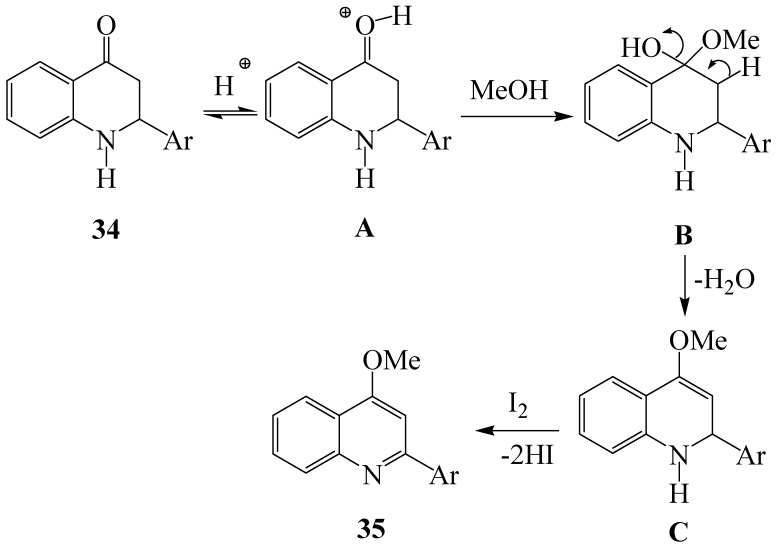
Iodine-promoted oxidative aromatization of 2-aryl-1,2,3,4-tetrahydroquinolin-4-ones.

The use of iodine as an effective oxidant to promote oxidative aromatization of α,β-unsaturated cyclic carbonyl compounds is not only limited to cyclohexenone derivatives. Molecular iodine in refluxing methanol has also been shown to effect oxidative aromatization of 2-aryl-1,2,3,4-tetrahydro-4-quinolones **34** to afford analogues of the naturally occurring 4-methoxy-2-phenylquinolines **35** with potential antimalarial and anti-tuberculosis activities (Scheme 14) [11]. The mechanism of this reaction is believed to involve initial attack of the protonated quinolone by methanol to generate a hemiacetal derivative. The latter would then undergo dehydration and subsequent oxidative aromatization by iodine to afford **35**.

### 2.3. Iodine/sodium ethoxide–mediated aromatization of cyclohexenone derivatives

**Scheme 15:**
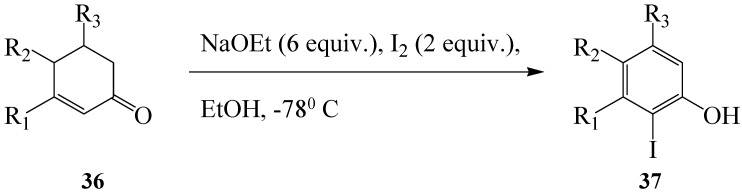
Iodine-sodium ethoxide–promoted aromatization of 2-cyclohexenone-4-carboxylates.

Hedge and coworkers, on the other hand, used iodine and sodium ethoxide to convert 2-cyclohexenone-4-carboxylates **36** into 2-iodophenols **37** (Scheme 15) [34]. The reaction was found to be favoured by the presence of electron withdrawing carboxyl group at the 4-position and to fail in the case of simple 2-cyclohexenones (R_1_,R_3_=H; R_2_=alkyl or phenyl) due to reduced acidity of the 4-methine or methylene protons.

**Scheme 16:**
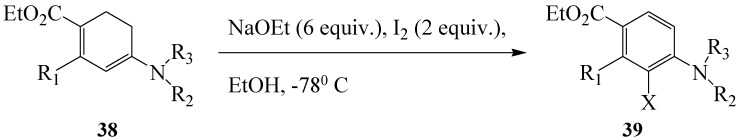
Iodine-sodium ethoxide–promoted aromatization of enamine derivatives.

The secondary enamines **38** (R_2_=H, R_3_=alkyl) with NaOEt (6 equiv.) and I_2_ (2 equiv.) in EtOH, -78 ºC, on the other hand, afforded the corresponding iodoanilines **39** (X=I) in 42–61% yield as single regioisomers (Scheme 16). Interestingly, the tertiary enamines **38** (R_2_,R_3_=alkyl, cycloalkyl) afforded the corresponding non-iodinated aromatic derivatives **39** (X=H) in 74–89% yield. The divergence in behavior of the tertiary enamines from those of 4-carboxy substituted cyclohexenones and their secondary enamine derivatives was attributed to different rates of iodination. It was proposed that the iodination of tertiary enamines is sufficiently slow to allow dehydroiodination of the incipient mono-iodo species to successfully compete with diiodination step and result in non-iodinated *N,N*-dialkylanilines. This proposal was further supported by oxidative aromatization of the tertiary enamine (NR_2_R_3_=morpholine) with iodine (1 equiv.) in the presence of triethylamine (2.5 equiv.) and 1,8-diazabicyclo[5.4.0]undec-7-ene (DBU) at room temperature to afford the corresponding *N,N*-dialkylaniline in 56 and 63% yields, respectively.

### 2.4. Iodine–mediated aromatization of Hantzsch ester 1,4-dihydropyridines 

**Scheme 17:**
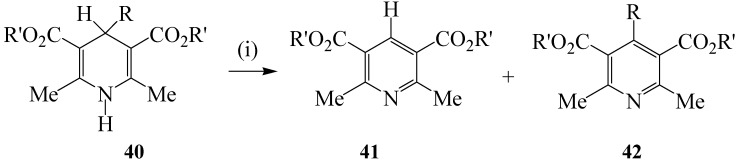
Iodine-promoted aromatization of 1,4-dihydropyridines under neutral and ultrasonic irradiation conditions.

Aromatization of Hantzsch ester 1,4-dihydropyridines using iodine in the presence of an alkali or organic base in methanol has been reported [35]. Iodine in refluxing acetonitrile is also reported to promote aromatization of the Hantzsch ester 1,4-dihydropyridines **40** to afford the corresponding pyridine derivatives **42** in high yields and regioselectivity in the absence or presence of ultrasound irradiation (Scheme 17) [36]. The 1,4-dihydropyridine derivative **40e** bearing a secondary alkyl group was found to undergo aromatization accompanied by dealkylation to afford the 4-unsubstituted pyridine derivative **41e** in excellent yield. The 4-unsubstituted **41** (**f** and **g**) and substituted derivatives **42** (**f** and **g**) were isolated as mixtures under both reaction conditions when 1,4-dihydropyridines bearing 2-furyl moiety on the 4-position were used as substrates. Although high yielding, aromatization of Hantzsch ester 1,4-dihydropyridines using iodine in methanol in the presence of a base [35] or under neutral conditions in acetonitrile [36] involve prolonged reaction times than that involving ultrasound (US) irradiation.

Aromatization of Hantzsch ester 1,4-dihydropyridines using iodine under conventional and ultrasonic irradiation was found to be superior to other methods that involve the use of strong oxidizing agents and severe conditions that require prolonged reaction times leading to low yields [36].

## 3. Conclusions

In summary, molecular iodine has established itself in chemical transformation as an efficient, readily available and easy-to-handle oxidizing agent to effect aromatization of α,β-unsaturated cyclohexenone derivatives and their heterocyclic analogues. The generality and brevity of iodine–mediated oxidative aromatization reactions and the accompanying high yields make this methodology a suitable alternative to metal–catalyzed aromatization of related derivatives. Furthermore, direct formation of phenol ethers using iodine–promoted oxidative aromatization avoids an additional step required to convert the hydroxyl compounds formed through metal-catalyzed or DDQ–mediated aromatization to the corresponding alkoxy derivatives.

## Figures and Tables

**Table:** 

	R	R_1_	R_2_	%Yield
**6a**	H	Me	H	87
**6b**	H	Ph	H	93
**6c**	H	n-Pr	H	86
**6d**	H	H	Me	83
**6e**	H	Ph	H	90
**6f**	TBS	Me	H	84

**Table:** 

	R_1_	R_2_	%Yield
**8a**	H	H	71
**8b**	H	Me	67
**8c**	Me	H	60
**8d**	Me	Me	50

**Reagents**: I_2_ (2 equiv.), MeOH, heat, 2-3 h.

**Table:** 

	R	%Yield of 11	%Yield of 12
**a**	H	12	22
**b**	Me	-	43
**c**	-CH=CH_2_	9	5

**Table:** 

	R_1_	R_2_	%Yield
**20a**	H	H	72
**20b**	Me	H	65
**20c**	H	Me	86

**Reagents**: (i) I_2_, MeOH, heat, 3h.

**Table:** 

	R_1_	R_2_	R_3_	Time (h)	%Yield 24	%Yield 25
**a**	H	Me	Me	17	89	-
**b**	H	Me	Et	15	70	-
**c**	H	Me	Bu	12	41	-
**d**	H	Et	Me	13	79	-
**e**	H	Ph	Me	14	28	48
**f**	H	OMe	Me	10	49	26
**g**	Me	Me	Me	20	63	9
**h**	Ph	Me	Me	20	50	12

*Reagents*: (i) I_2_ (2 equiv.); R”OH, heat, 12-20 h.

**Table:** 

	R_1_	R_2_	%Yield of 27	%Yield of 28
**a**	H	Me	11	66
**b**	Me	Me	5	59
**c**	Ph	Me	6	52
**d**	H	Et	12	45

**Table:** 

	R_1_	R_2_	R_3_	Time (h)	%Yield of 30	%Yield of 31
**a**	H	H	Ph	7	64	-
**b**	H	Me	Ph	1	72	-
**c**	H	Me	4-ClC_6_H_4_	3	88	-
**d**	Me	Me	4-ClC_6_H_4_	2	81	-
**e**	Me	Et	Ph	1	82	-
**f**	Me	Me	(Me)_2_CH	1	64	18
**g**	H	Me	(Me)_2_CH	1	64	6

**Table:** 

	Ar	%Yield
**35a**	C_6_H_5_	73
**35b**	4-FC_6_H_4_	80
**35c**	4-ClC_6_H_4_	91
**35d**	4-BrC_6_H_4_	65
**35e**	4-MeOC_6_H_4_	90
**35f**	4-NO_2_C_6_H_4_	60

*Reagents*: (i) I_2_ (2 equiv.), MeOH, heat, 2h.

**Table:** 

	R_1_	R_2_	R_3_	%Yield
**37a**	H	CO_2_Me	Me	66
**37b**	H	CO_2_Et	CF_3_	86
**37c**	H	CO_2_Et	CO_2_Et	77
**37d**	H	CO_2_Et	2-Thienyl	61
**37e**	H	CO_2_Me	2-Furyl	50
**37f**	Me	CO_2_Et	H	78
**37g**	CF_3_	CO_2_Et	H	65
**37h**	Ph	CO_2_Et	H	64

**Table:** 

	R	R’	Time (h)^a^	Time (h)^b^	%Yield^a^	%Yield^b^
**42a**	Ph	Et	5	15	96	98
**42b**	Ph	Me	6	20	94	96
**42c**	3-NO_2_C_6_H_4_	Et	7	30	95	95
**42d**	2-NO_2_C_6_H_4_	Me	4	40	91	94
**41e**	(Me)_2_CH	Me	1	5	96	98
**42f+41f**	2-Furyl	Et	5	10	60+40	70+30
**42g+41g**	2-Furyl	Me	5.5	15	65+35	70+30
**42h**	4-(MeO)C_6_H_4_	Et	3	40	93	94
**42i**	4-(MeO)C_6_H_4_	Me	4	45	93	94
**42j**	4-MeC_6_H_4_	Et	4	30	95	97
**42k**	4-MeC_6_H_4_	Me	4	35	95	96
**42l**	4-ClC_6_H_4_	Me	4	25	94	94
**42m**	4-Hydroxy-3-methoxyphenyl	Et	1	20	97	98

*Reagents*: (i) I_2_ (2 equiv.), CH_3_CN, heat; ^a^ I_2_ (2 equiv.), CH_3_CN, heat; ^b^ US irradiation.
